# Supplementary material for: Changes in Soil Characteristics, Microbial Metabolic Pathways, TCA Cycle Metabolites and Crop Productivity following Frequent Application of Municipal Solid Waste Compost
Source: Plants (Basel). 2022 Nov 18;11(22):3153. doi: 10.3390/plants11223153 (PMC9695376; doi:10.3390/plants11223153)
Supplement: Supplementary file 1 [file plants-11-03153-s001.zip › plants-2001119-supplementary.pdf]

Table S1. Climate conditions in Brandon, Manitoba during the growing season between May to August 2015 to 2019, and 30-year average (1981-2010) (Government of Canada, 2020).

Monthly temperature (°C)									
Year	May		June		July		August		Annual mean
	Maximum	Minimum	Maximum	Minimum	Maximum	Minimum	Maximum	Minimum	
2019	17.3	0.4	23.9	8.7	25.7	11.8	24	8.8	15.1
2018	23.2	4.5	25.7	11.5	26.2	10.8	26.8	7.9	17.1
2017	18.8	3.4	22.9	8.8	26.7	11.2	25.3	8.8	15.7
2016	21	4.9	23.9	10	25.1	12	25.3	9.5	16.5
2015	18.4	1.8	24.3	9.6	26.1	12.2	26.2	9.8	16.1
1981-2010	17.8	3.4	22.4	9.3	25.2	11.7	24.9	10.4	15.6

Total precipitation (mm)					
Year	May	June	July	August	Annual Mean
2019	42.9	78.2	48.2	27.6	49.2
2018	24.0	85.2	52.6	25.4	46.8
2017	21.4	70.6	36	37.4	41.4

2016	55.4	106.4	98	0	65.0
2015	48.6	37.4	64.8	50.4	50.3
1981-2010	59.1	80.7	73.4	65.9	69.8

Table S2. Five-year average chemical elements concentration of municipal solid waste compost.

Property	Value	Element	Value (%)	Element	Value (%)
pH	8.7	Nitrogen	1.06	Manganese	118
Lime index	6.9	Phosphorus	573	Iron	141
Soluble salt (mS/cm)	4.9	Potassium	5359	Copper	2.0
Available OM[1]	14.7	Magnesium	836	Boron	3.5
CEC[2] (meq/100 g)	55.2	Calcium	6187	Sodium	833
Nitrate-nitrogen (mg/kg)	58.0	Sulphur	171	Mg:K	1:1
		Zinc	17.8	Ca:Mg	4:1

[1]OM, percentage available organic matter content; and [2]CEC, cation exchange capacity.

Table S3. Summary of deblur output samples.

Metric	16S rRNA	ITS2
Deblur output statistics		
Number of samples	45	45
Number of features	55,483	1,281
Total frequency	892,582	188,044
Minimum frequency	7,570	280
1st quartile	16,102	2,032
Median frequency	19,867	3,701
3rd quartile	23,740	5,391
Maximum frequency	33,775	14,051
Mean frequency	19,835	4,178
Statistics after quality control and normalization		
Number of samples after normalization	43	39
Number of features after quality control and normalization	1,768	721
Total frequency after quality control and normalization	273,050	46,254
Frequency per sample after quality control and normalization	6,350	1,186

Table S4. Relative abundances of major soil microbial taxa.

Taxa	Relative abundances
**Fungi**	
Basidiomycota<br>Tremellomycetes – 24%<br>Agaricomycetes – 11%<br>Ustilaginomycetes – 6%<br>Other – <1%	42%
Mortierellomycota<br>Mortierellomycetes – 41%<br>Other – <1%	41%
Ascomycota	5%
Chytridiomycota	4%
Others	8%
**Bacterial**	
Actinobacteria<br>Actinobacteria – 17%<br>Rubrobacteria – 7%<br>Thermoleophilia – 6%<br>MB-A2-108 – 3%<br>Other – 1%	35%
Proteobacteria<br>Alphaproteobacteria – 14%<br>Gammaproteobacteria – 5%<br>Deltaproteobacteria – 5%<br>Other - <1%	23%
Acidobacteria<br>Subgroup 6- 7%<br>Blastocatellia (Subgroup 4) – 2%<br>Other – 1%	11%
Chloroflexi<br>Anaerolineae – 6%<br>Chloroflexia – 2%<br>Other – 3%	11%
Bacteroidetes<br>Bacteroidia – 5%<br>Other - <1%	5%
Firmicutes	3%
Gemmatimonadetes	3%
Other	6%

Table S5. Variations in soil sample microbial groupings explained by weighted UniFrac beta-diversity distances.

Grouping (subset)[a]	ITS (R^2)	16S rRNA (R^2)
Soil treatment combined	0.085*	0.442***
Annual versus No compost application	0.050ns	0.507***
Biennial versus No compost application	0.080*	0.257***
Annual versus Biennial compost application	0.067*	0.249***

Adonis tests were used to assess whether beta-diversity is related to sample groupings, 999 permutations; R^2, coefficient of correlation; *, *** are significant at $P < 0.05$ and $P < 0.001$, respectively; ns, not significant at $P > 0.05$.

Table S6. Concentrations of essential and beneficial chemical elements in edible portions of green beans (*Phaseolus vulgaris* cv. Golden Wax), beets (*Beta vulgaris* cv. Detroit Supreme) and lettuce (*Latuca sativa* cv. Grand Rapids) as affected by frequency of application of municipal solid waste compost.

	Green beans (mg/kg)			Beets (mg/kg)			Lettuce (mg/kg)			
Chemical elements	Annual	Biennial	Control	Annual	Biennial	Control	Annual	Biennial	Control	CV[1]
Total nitrogen	1600	700	600	<100	<100	<100	4900	1200	200	0.81
Phosphorus	4060	4040	3650	2910	2990	2000	4200	4170	2570	3.05
Potassium	34850	25000	23700	34700	25600	21300	96600	67600	37800	5.75
Magnesium	2395	2650	2360	2220	2120	2210	4600	4980	5930	1.95
Calcium	4090	4000	4310	1810	1590	2270	12400	13100	13600	1.21
Boron	19.2	18.6	23.0	19.4	18.0	18.3	23.2	23.8	24.6	0.22
Iron	62	65	74	43	39	36	364	259	258	1.29
Manganese	19	19	20	33.9	29.6	30.7	91	95.8	81.9	0.91
Molybdenum	7.39	7.55	3.26	0.91	0.65	0.52	4.13	4.34	2.12	0.11

Cobalt	0.12	0.13	0.11	0.03	0.03	0.04	0.17	0.13	0.15	0.02
Sodium	40	30	40	7830	5370	2530	3490	2050	2020	2.00
Chromium	1.80	0.3	0.6	0.4	0.3	0.2	3.7	3.5	3.6	0.01
Copper	4.7	5.7	5.0	8.5	6.0	4.9	5.9	6.3	7.3	0.32
Nickel	1.6	1.5	1.3	1.2	1.3	0.2	1.9	5.1	2.6	0.02
Zinc	20.6	21.5	18.9	30	21.8	18.4	37.1	30.2	31.9	1.91

[1]Nitrate and nitrite as total nitrogen; CV (%), coefficient of variation (source: Abbey et al., 2021).
